# An automated multi-layer perceptron discriminative neural network based on Bayesian optimization achieves high-precision one-source single-snapshot direction-of-arrival estimation

**DOI:** 10.1038/s41598-024-60798-w

**Published:** 2024-05-05

**Authors:** Bin Zhang, Jiawen He, Peishun Liu, Liang Wang, Ruichun Tang

**Affiliations:** 1 Department of Computer Science and Technology, Ocean University of China, Qingdao, 266100 China; 2 Department of Marine Technology, Ocean University of China, Qingdao, 266100 China

**Keywords:** Computer science, Physical oceanography

## Abstract

This paper proposes an innovative global solution which is a pioneering work applying automated machine learning algorithms to remarkable precision sparse underwater direction-of-arrival (DOA) estimation that views the subaquatic sparse-sampling DOA estimation problem as a classification prediction task. The proposed solution, termed automated multi-layer perceptron discriminative neural network (AutoMPDNN), is built upon a Bayesian optimization framework. AutoMPDNN transforms sparsely sampled time-domain signals into the complex domain, preserving essential components in a one-source single-snapshot scenario. Leveraging Bayesian optimization principles, the algorithm embeds necessary hyperparameters into the loss function, effectively defining it as a maximum likelihood problem using the upper confidence bound function and incorporating sparse signal features. We also explore the model space architecture and introduce variants of AutoMPDNN, denoted as AutoMPDNNs_ln (n = 2,3,4). Through a series of plane wave simulation experiments, it is demonstrated that AutoMPDNN achieves the highest prediction performance for one-source single-snapshot scenarios compared to classical DOA estimation algorithms that incorporate sparse representation approaches, as well as contemporary deep learning DOA methods under varying conditions.

## Introduction

In diverse oceanic acoustic environments, DOA estimation plays a crucial role in signal processing and underwater target detection, serving as a fundamental technique for extracting information regarding the direction of acoustic sources from array receivers^1–3^. Conventional beamforming (CBF) algorithms^4^ utilize the combination of multiple signals to achieve coherent and incoherent interference, suppressing interference signals from non-target directions while enhancing the acoustic signals from the target direction, resulting in the formation of beam target direction signals. However, they exhibit poor robustness and lower resolution in complex interference environments, situations involving fast-moving or non-static targets, spatial sampling limitations, and similar conditions. The minimum variance distortionless response (MVDR) algorithm^5^ enhances the gain of the receiving array by minimizing the average output power through the introduction of adaptive complex weighting into the array receiver’s signal expression. This improvement bolsters the robustness in noisy environments and the signal interference resistance of conventional beamforming algorithms. The Generalized Cross-Correlation (GCC) method^6^ employs the generalized cross-correlation functions of signals to estimate time delays. Compared to CBF algorithms, it offers lower computational complexity and can swiftly estimate the arrival angles of signal sources, regardless of signal coherence. The Multiple Signal Classification (MUSIC) algorithm^7^ decomposes the observation space of non-coherent source signals into signal and noise subspaces, leveraging the invariance of the arrival angles of wavefronts between array elements with finite-time snapshots. The ESPRIT algorithm^8^ and Root-MUSIC algorithm^9^, as variants of the MUSIC algorithm, respectively leverage the rotational invariance of spatial arrays and root-finding processes to reduce the complexity of the MUSIC algorithm. The MUSIC algorithm and its variants break through the Rayleigh resolution limit of CBF, achieving high resolution in ideal signal-to-noise ratio (SNR) conditions with non-coherent sources and a known number of signal sources. The aforementioned traditional DOA estimation approaches often require prior information or pre-knowledge of target direction and suffer from issues such as high computational complexity, sensitivity to data quality, and a lack of adaptability to changes and data diversity.

However, in highly dynamic and short-duration scenarios, it is often impossible to obtain sufficient independent and identically distributed snapshot data, leading to a drastic decline in the accuracy of traditional DOA estimation algorithms. Even under conditions of sparse snapshots and finite array element reception, the aforementioned classical DOA estimation methods are unable to accurately identify the directional information of incoming waves. Hence, the hot topic in current DOA estimation research revolves around how to extract highly usable features from sparse data received within a limited time frame and design efficient, lightweight algorithm models^10–14^. With the introduction and maturation of compressive sensing theory^15^, sparse signal reconstruction has broken through the bottleneck of the Nyquist sampling theorem, overcoming the problem of DOA estimation under sparse sampling. Compressive sensing-based DOA estimation methods discretize the azimuth angles of wave arrivals into a grid and use the steering vector as the sensing matrix, transforming the DOA estimation problem into a sparse signal recovery problem^16^. The Matching Pursuit (MP) algorithm^17^ iteratively selects the most matching atoms from an overcomplete dictionary in the Hilbert space to approximate the signal residue, where the positions of non-zero elements represent the DOA targets. The Orthogonal Matching Pursuit (OMP) method^18^ requires regularization and orthogonal processing of atoms during the decomposition process. The OMP algorithm improves the orthogonality between the residue and atom projections in the iterative process of the MP algorithm, enhancing both the computational speed and accuracy of the MP algorithm. The Regularized Orthogonal Matching Pursuit (ROMP) algorithm^19^ abandons the greedy principle of the OMP in atom selection, instead selecting a regularized vector with the maximum absolute inner product with the residue as the atom set at each iteration, thereby enhancing the robustness of the OMP algorithm. The Compressive Sampling Matching Pursuit (CoSaOMP) approach^20^ builds upon the ROMP algorithm by updating k-column residues at each iteration, eliminating some minimally correlated atoms. This improves the efficiency and convergence speed of the ROMP algorithm. In contrast to the greedy approaches mentioned above, the *l*1SVD algorithm^21^ employs singular value decomposition and minimizes the *l*1 norm principle, transforming the nonlinear convex optimization objective function into a second-order cone programming problem. This method calculates the angles of sparse signal arrivals by grid-searching the azimuth angles. The *l*1SVD algorithm achieves dimension reduction in feature selection and effectively suppresses environmental noise interference, enhancing the robustness of sparse target signals against interference. Furthermore, the *l*1SRACV algorithm^22^, compared to *l*1SVD, does not require prior knowledge of the number of sources. By solving covariance matrix operations, it achieves high accuracy in direction finding under low snapshot and low SNR conditions. Nonetheless, this algorithm increases spatial computational complexity and has higher time complexity. Although compressive sensing algorithms address sparse snapshot DOA estimation problems, they still require substantial prior knowledge and expert experience as guidance. In recent times, as statistics continue to advance, machine learning has been challenging traditional approaches in various fields such as image processing, natural language, and signal processing, including underwater acoustic source localization^29–32^. Based on machine learning, A novel method integrates compressive sensing and machine learning methods^23^, referred to as NN, taking the normalized impinging signal and the ratio between adjacent beamforming outputs as input. The NN achieves predictions of DOA estimation and frequencies with lower computational complexity and stronger robustness using a Uniform Linear Array in a single snapshot. This research demonstrates the effectiveness of neural network in source localization in complex and varied environments, especially in the case of sparse samples in a training set. In another investigation, a multi-layer nonlinear deep feedforward neural network (FNN) model^24^ designs for DOA estimation. FNN takes the received array signals as input and redefines, traditional beamforming as a real-valued linear inverse problem in weight space. It updates neural network weights and biases through both exhaustive and random trainings. The network’s hidden and output layers are then used to predict the arrival direction of sources. This method achieves up to 95.9% accuracy in the DOA estimation for two sparse sources with a single snapshot, proving that feedforward neural networks offer strong accuracy and robustness in estimating the DOA of sparse signals in complex noise and interference environments. This work further explores 2D convolutional neural networks, using the sample covariance matrix as dual-channel input. Leveraging the local connectivity and weight sharing features of CNNs, a three-layer CNN is designed to achieve an accuracy of 74.1% in a single snapshot. Compared to traditional shallow convolutional neural networks, deeper ResNet^25^ based on residual structures(2D) are designed for DOA estimation. This research utilizes the covariance matrix of single snapshot sample data as input features. By leveraging ResNet with layer-wise residual blocks, it achieves a 90.1% accuracy in DOA estimation for dual-target sources at an SNR of 20 dB. Further, Deep-MLE^26^ integrates 1D ResNet with Maximum Likelihood Estimation (MLE) methods. This study combines physical constraints with deep learning networks. Compared to using 2D ResNet alone, the computational complexity is reduced from 5.1$\times 10^{6}$ to 1.68$\times 10^{6}$ while maintaining consistent accuracy. Yang et al. proposed a DeepDOA super-resolution algorithm^27^ that combines DNN and CNN to reconstruct the spatial spectrum representation. DeepDOA is capable of learning key features from raw single snapshot data for real-time inference, exhibiting performance similar to sparse representation algorithms but with lower computational complexity. Zheng et al. designed a deep neural network^28^ with fewer parameters to fit the minimum power distortionless response (MPDR) beamformer features. They demonstrated the superiority and real-time capability of deep learning in single snapshot scenarios across different SNR environments. Those studies demonstrate the potential of deep neural network models in sparse single snapshot scenarios. Though the aforementioned studies robustly demonstrate the effectiveness and robustness of deep learning on sparse single snapshot signals, the selection of hyperparameters in deep learning algorithms will directly affect the generalization performance of the entire model. These studies did not design the model from the perspective of global convergence, but instead fixed the training model based on limited hyperparameters. This often leads to models with local consistency, which are prone to getting trapped in local optima.

In this work, we depart from convolutional neural networks with local connections and weight sharing. Instead, we introduce a novel lightweight AutoMPDNN designed for single-snapshot sparse data and optimized for smaller hydrophone arrays using Bayesian optimization. AutoMPDNN uses supervised learning with labels in the [${0}^{\circ }$,${180}^{\circ }$] range to achieve end-to-end DOA estimation. In response to the analysis presented above, this paper introduces the following contributions:

- Addressing the challenge of single-snapshot sparse data, we reconstruct signals to extract crucial feature information. We have designed a lightweight, multi-layer perceptual discriminative network model (MPDNN) based on supervised learning using multi-layer perceptron network model.
- We employ a Bayesian optimizer to design an automated deep learning optimization strategy that constrains the MPDNN, that is AutoMPDNN. This strategy automates the optimization of the model by treating hyperparameters as penalty terms in the loss function.
- Through simulation experiments in various SNR scenarios, we demonstrate the limitations of traditional DOA methods and showcase the accuracy and real-time performance of AutoMPDNN compared to other deep learning approaches.

## Methods

In this chapter, we have developed AutoMPDNN which based on a multi-layer perceptron network model. First, we introduce the input data for the model, specifically the signal model construction and feature preprocessing. Next, we provide an overview of the proposed MPDNN architecture. Finally, we introduce the automated hyperparameter search algorithm based on Bayesian optimization and its extensions, AutoMPDNN and AutoMPDNNs, which are designed to optimize the training processes of MPDNN and MPDNNs.

### Reception and reconstruction of array signals

**Figure 1:**
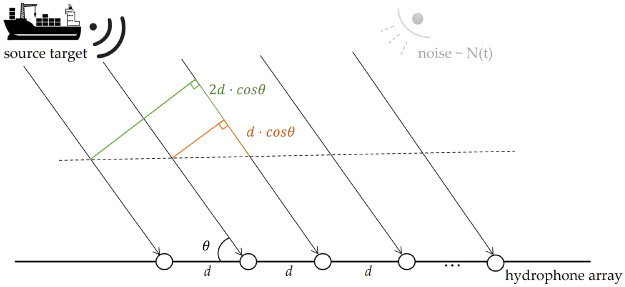
Narrowband far-field horizontal linear array signal reception model.

In the narrowband far-field scenario of a horizontal linear array, as shown in Fig. 1, a planar wave emitted by a one-source is received by an array of *M* horizontal elements along the x-axis, with an inter-element spacing of *d*. There is a time delay difference of $d\cdot cos\theta /c$ between adjacent array elements in the same subarray, where *c* means sound velocity. The angle $\theta$, which represents the angle between the source and the horizontal array, ranges from $\theta \in [{0}^{\circ },{180}^{\circ }]$. The temporal signals *X*(*t*) received by the array at discrete time snapshots *t* can be expressed as shown in Eq. (1), which represents the signal reception model for the array.

1 $$\begin{aligned} X(t)=AS(t)+N(t) \end{aligned}$$

More specifically, we assume that the source signal *S*(*t*) is a single-frequency cosine wave and its frequency is denoted as *f* , as shown in Eq. (2).

2 $$\begin{aligned} S(t) = 10^{({SNR/20})}cos{({2\pi ft})} \end{aligned}$$

The steering vector *A* received by *M* arrays of *k* acoustic source at equal intervals *d* is defined as shown in Mat. (3).

3 $$\begin{aligned} A = \begin{bmatrix} {~e^{j0\varphi _{1}}~~~~~~{~~~~~~~~e}^{j0\varphi _{1}}} &{} \cdots &{} e^{j0\varphi _{1}} \\ \vdots &{} \ddots &{} \vdots \\ {{~~~e}^{j{({M - 1})}{\varphi }_{1}}~~~~~~e^{j{({M - 1})}{\varphi }_{2}}~~~} &{} \cdots &{} e^{j{({M - 1})}{\varphi }_{k}} \end{bmatrix} \end{aligned}$$

In this regard, $\varphi _{i} = \left( {2\pi d} \right) cos\theta _{i}/\lambda$, $\lambda$ is wave length and $\lambda =c/f$, where $i\in [1,k]$. Gaussian white noise is represented as $N(t)=[n_1 (t),n_2 (t),\ldots ,n_M (t)]$. By employing the Euler’s formula $e^{ix} = cosx + isinx$, we can substitute it into the signal reception Eq. (1), resulting in the following transformation:

4 $$\begin{aligned} \begin{aligned} cos(\omega t)*e^{j\varphi }&=[Re\{ e ^{j\omega t} \} + Re\{ e ^{- j\omega t} \}]*[cos(\varphi ) + jsin(\varphi ) ]\\ {}&= [Re\{ e ^{j\omega t} \} cos(\varphi ) - Im\{ e ^{j\omega t} \} sin(\varphi ) \\ {}&\quad + j[Re\{ e ^{j\omega t} \} sin(\varphi ) + Im\{ e ^{j\omega t}\} cos(\varphi )]]\\ {}&\quad + [Re\{ e ^{- j\omega t} \} cos(\varphi ) - Im\{ e ^{- j\omega t} \} sin(\varphi ) \\&\quad + j[Re\{ e ^{- j\omega t} \} sin(\varphi ) + Im\{ e ^{- j\omega t} \} cos(\varphi )]]\end{aligned} \end{aligned}$$

In this context, $\omega = 2\pi f$ represents the angular frequency. In the above equation, due to the complex conjugate nature of $e^{j\omega t}$ and $e^{-j \omega t}$, according to the properties of complex conjugates, the equation can be rewritten as follows:

5 $$\begin{aligned} cos(\omega t)\left. *e^{j\varphi } = 2Re\{ e \right. ^{j{({\omega t + \varphi })}} \} \end{aligned}$$

From Eq. (5), it can be observed that the azimuthal information of the ranged snapshot signal received by the array in the complex domain is related to the phase difference of the steering vectors. According to the theory of short-time Fourier transform^33^, any signal can be decomposed into complex amplitudes varying with time and frequency within a short unitary time period. Substituting Eqs. (5) and (2) into equation (1) yields the reconstruction expression for the array-received signal within a specified snapshot. Therefore, we can reconstitute the frequency *f* expression for the array input signal data as below:

6 $$\begin{aligned} x_{m}(f) = {\sum _{l = 1}^{k}[{a_l}}e^{j{({m - 1})}\varphi _{l}} ]+ n_{m}(f),~m \in [1,M]\end{aligned}$$

Here, $a_l$ represents the amplitude of the *l*-th signal, and $n_m (f)$ denotes the Gaussian white noise received by the *m*-th array element. Then, the entire time-domain signal received by the array can be reconstructed as follows:

7 $$\begin{aligned} {X(f) = \big [x_{0}(f),{~x}_{1}(f),\ldots ,~x }_{m}(f) \big ]\end{aligned}$$

Within this context, $x_{i}(f) = \big [x_{i}^{R},x_{i}^{I} \big ]^{T}$, where $i \in [0,m]$, $x_{i}^{R}$ and $x_{i}^{I}$ embody amplitude and phase characteristics. The reconstructed array *X*(*f*) receive sequence now contains both amplitude and phase information, with dimensions [2*M*, *L*], where *L* is the number of sampling points. When *L* is set to 1, the reconstructed single-snapshot signal exhibits a certain level of sparsity within a finite number of array elements.

### Multi-layer perceptron discriminative neural network

**Figure 2:**
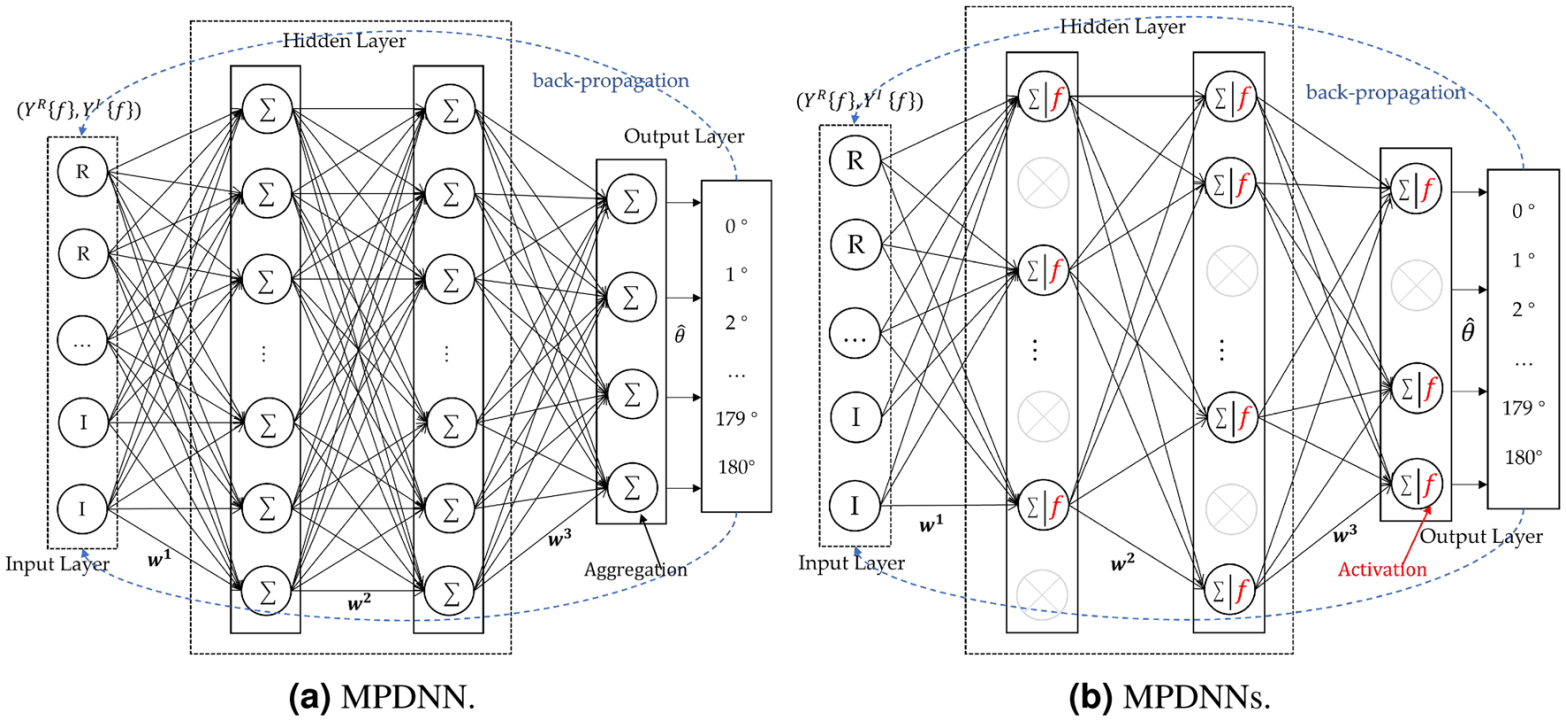
MPDNN and MPDNNs, where $( Y ^{R}\left\{ f \right\} ,Y^{I}\left\{ f \right\} ) = {X(f)}$ , with details of *X*(*f*) provided in Eq. (7).

The attention mechanism^34^ is employed in various deep learning applications to acquire contextual weight information through different weighted scoring functions, enhancing the feature selection focus of classification models. Building upon the attention mechanism, the Transformer^35^ introduced a multi-head self-attention mechanism. The encoder-decoder structure allocates weights to the value vectors based on the similarity between query vectors and key vectors to generate weighted outputs. The Transformer enables models to establish relationships between different positions for better capturing dependencies within sequences. Self-attention computes feature-weighted updates for different positions across all input vector locations. For an input sequence with *N* positions and *D* dimensions, as well as an attention mechanism with *H* attention heads, the computational complexity is $O(N^2D+D^2HN)$. This implies that the Transformer exhibits quadratic growth in computational complexity as the sequence length increases and overlooks potential correlations between different samples. Perceptron models, as a type of nonlinear classifier, are commonly employed in deep neural network classification tasks. In contrast to traditional two-layer perceptron models, multi-layer perceptrons exhibit enhanced nonlinear fitting capabilities^36^. The External Attention model^37^ has demonstrated remarkable results comparable to Transformer using only two cascaded linear layers. The emergence of External Attention underscores the potent nonlinear fitting capacity of fully connected layers. Inspired by External Attention, we utilize the sparse signals extracted as discussed in previous section as input data. Two end-to-end lightweight models are designed, MPDNN and MPDNNs, with the multi-layer perceptron network model as the backbone. The model treats DOA estimation as the optimization target for a multi-class classification task, thereby transforming the DOA estimation problem into a sparse parameter learning problem, denoted as $\{F(w)|X(f,\theta )\}$. The model structure is depicted in Fig. 2.

First, we concatenate the real part and the imaginary part to obtain a sequence of dimensions $R^{2M\times L}$, denoted as $( Y ^{R}\left\{ f \right\} ,Y^{I}\left\{ f \right\} )$. This facilitates the handling of complex-valued input data. After several layers of parameter learning and propagation through feedforward perceptron networks, the expression of the high-order speech signal at the output layer is as Eq. (8).

8 $$\begin{aligned} O = ((Y ^{R}\left\{ f \right\} ,Y^{I}\left\{ f \right\} )w^{p - 1} + b^{p - 1})w^{p} + b^{p} \end{aligned}$$

In this context, *p* represents each hidden layer, and *w* and *b* represent the parameters and bias terms. From equation (8) it is evident that a multi-layer perceptron exhibits a high-degree linear affine transformation capability. However, as the number of network nodes increases and the network depth varies, deep learning models often encounter the issues of vanishing gradients or exploding gradients. To address these challenges and enhance the classification performance of the network, we introduce a non-linear transformation mechanism through the use of an activation function, preventing such problems. In this case, we employ the rectified linear unit (Relu) activation function^38^ to enhance the model’s one-sided inhibition and wide excitatory boundary capabilities. The expression for the Relu activation function is as follows:

9 $$\begin{aligned} Relu\left( O^{p} \right) = max\left( 0,O^{p} \right) \end{aligned}$$

We introduce dropout^39^ to enhance the robustness of MPDNN, named multi-layer sparse discriminative neural network (MPDNNs). MPDNNs involves allowing the dropout rate of neurons in MPDNN to follow a $\delta ~Bernoulli(q)$ distribution, thereby improving the network’s regularization capacity. Combining Eqs. (8) and (9), the output of MPDNNs can be expressed as:

10 $$\begin{aligned} Y\left( \hat{w} \right) = (1 - q)Relu( { w^{p}( {( Y^{R}\{ f \},Y^{I}\{ f \}})w^{p - 1} + b^{p - 1}) + b^{p}}) \end{aligned}$$

$Y\left( \hat{w} \right)$ represents the probability value of the predicted azimuth angle $\theta$. If *q* equals 0, MPDNNs are equivalent to MPDNN. Finally, we utilize the softmax algorithm to output the network’s classification results. During the reverse weight update process in model learning, the optimization of the spatial objective for the entire algorithm is defined by minimizing the deviation between the predicted angle and the true angle. The algorithm’s convergence is framed as a function optimization problem, using the information entropy of the true azimuth angle $\theta$ and the kullback-leibler divergence of the predicted angle $\hat{\theta }$ difference as the loss function for global computation. The loss function under N different *batchsizes* is defined as shown in Eq. (11).

11 $$\begin{aligned} J\left( \hat{w} \big | Y,\theta \right) = - \frac{1}{n}{\sum \limits _{n = 1}^{N}{{I\left( {\hat{\theta } = \theta } \right) }}} \cdot log\left( \frac{Y_{n}^{\hat{\theta }}\left( \hat{w} \right) }{\sum \limits _{\hat{\theta }= 0}^{180}{Y_{n}^{\hat{\theta }}\left( \hat{w} \right) }} \right) \end{aligned}$$

### Automatically learning and solving the optimization model based on the bayesian theory

Although MPDNN exhibits excellent nonlinear fitting capabilities, fine-tuning its parameters for network design poses an extremely complex engineering challenge. Random forests^40^ apply the ensemble learning concept, treating each classifier as a decision tree unit. Different decision trees are considered weak classifiers, and the optimal solution is selected using a weighted voting mechanism. Ensemble learning combines the basic models of weak learners, collectively improving the model’s pre-dictive capabilities. Inspired by AutoML^41^, AutoML incorporates the concept of en-semble learning, defining different neural network parameter spaces as decision trees. It iteratively identifies the optimal decision model by designing network structure selec-tion methods and hyperparameter search strategies. Drawing inspiration from AutoML, AutoMPDNN approaches neural node and hyperparameter optimization as an auto-mated global search task, aiming to minimize model loss as a constraint. Weight learning in the proposed network and hyperparameter search are treated as a multi-task optimization problem. Each search space acts as a decision tree, and the optimal sub-tree is selected independently through a voting mechanism to find the optimal hyperpa-rameters for AutoMPDNN. Therefore, equation (11) can be redefined as an optimization problem under the constraint of hyperparameters as Eq. (12).

12 $$\begin{aligned} L\left( \hat{w} \big | Y,\theta ,\eta \right) = J\left( \hat{w} \big | Y,\theta \right) + Q{(\eta )} \end{aligned}$$

Here, $Q(\eta )$ represents the automated hyperparameter constraint space, where $\eta$ is the hyperparameter set of AutoMPDNN. According to Eq. (12), $Q(\eta )$ can be transformed into the following constraint:

13 $$\begin{aligned} \eta ^{*} = {\underset{\eta \in \varrho }{argmin}{Q{(\eta )}}} \end{aligned}$$

$Q(\eta )$ is evidently a non-convex function, and solving $Q(\eta )$ using a grid-based algorithm, created through the parameter search space and variable generation process, will require relatively expensive computational resources. Based on Bayesian optimization theory, Eq. (12) can be reformulated to find the maximum likelihood estimate of $\eta$ under the prior conditions of $\hat{w}$.

14 $$\begin{aligned} P\left( \hat{w} \big | {L(\eta )} \right) = \frac{P\left( \hat{w} \right) P\left( {L(\eta )} \big | \hat{w} \right) }{P\left( {L(\eta )} \right) } \end{aligned}$$

The single-modal sine/cosine waves decomposed from equation (6) represent periodic signals. The kernel function of the Gaussian process determines the correlations between different input points. Therefore, we utilize the Gaussian kernel function, $GP(\cdot )$, to model this periodicity as a prior for equation (12), denoting it as $f_l \sim GP(\cdot )$.

15 $$\begin{aligned} f_{l}\left( {\eta _{1},\eta _{2},\ldots ,\eta _{sizeof{(\varrho )}}} \right) = N\left( {\mu (\eta ),\Sigma \left( {\eta _{i},\eta _{j}} \right) } \right) \end{aligned}$$

In this context, $\eta _i$ represents the *i*-th coordinate point, $\mu (\eta )$ stands for the mean function, and $\Sigma \left( \eta _{i},\eta _{j} \right)$ denotes the covariance function. We employ the *UCB* strategy function^42^, denoted as $S_{ucb}$, to select the next optimal sampling point based on the posterior distribution, which is then added to the collection *D*. $S_{ucb}$ explores the global optimal solution region when $\sigma (\eta )$ is high and exploits local optimal solutions of $\eta$ within a relatively high $\mu (\eta )$. To strike a steady balance between exploitation and exploration, we consider the training set as empirical accumulation while the validation set is more aligned with the predictive distribution. Therefore, we introduce a balancing factor, *ka*, which is calculated as $ka=sizeof(Validating set)/ sizeof(Training set)$.

16 $$\begin{aligned} S_{ucb} = \mu (\eta ) + ka \cdot \sigma (\eta ) \end{aligned}$$

Then, the process iteratively updates the prior knowledge and stores the current optimal hyperparameter solution until all job scheduling tasks are completed. Finally, the best hyperparameter ${~\eta }^{*}$ is chosen from all the saved scheduling jobs. Throughout the entire Bayesian optimization process, a search is conducted in the hyperparameter space for MPDNN, including the layer nodes *l*1 and *l*2, the learning rate during the neural network convergence process, the random dropout rate for the dropout, and different batch sizes to select the optimal global loss function minimum. Simultaneously, data normalization is employed to balance the feature scales of different attributes of the signal’s real and imaginary parts, which accelerates the convergence speed of model parameter search and reduces the instability caused by dimension scale bias.

17 $$\begin{aligned} {Y_{R}^{\prime }\left\{ f \right\} },{Y_{I}^{\prime }\left\{ f \right\} } = \frac{X(f) - min\left( {X(f)} \right) }{max\left( {X(f)} \right) - min\left( {X(f)} \right) } \end{aligned}$$

where $X(f){=( Y}^{R}\{ f \},Y^{I}\{ f \} )$, denoting $Y=({{Y_{R}^{\prime }\{ f \}},{Y_{I}^{\prime }\{ f \}}})$. The entire process and algorithm principles of automated hyperparameter learning are depicted in Algorithm 1, *IMP* means inited AutoMPDNN model with indicated parameters.

**Algorithm 1:**
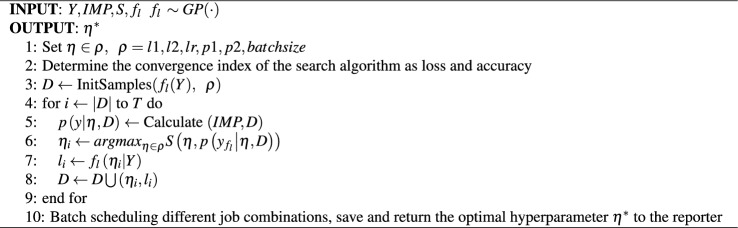
AutoMPDNN hyperparameter space search algorithm based on Bayesian optimizer.

### Training process

**Figure 3:**
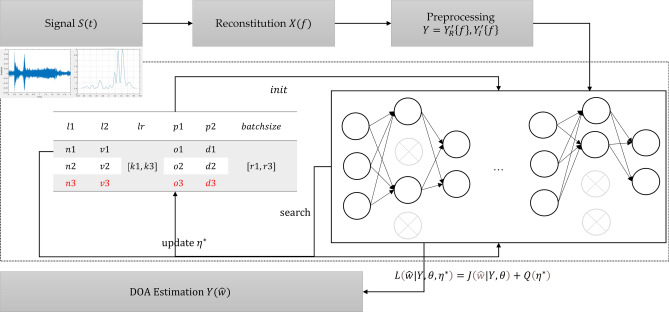
AutoMPDNN training and estimation workflow.

AutoMPDNN is trained using a supervised learning approach. The entire dataset used in this experiment consists of simulated data. The training, validation, and testing processes of the entire network follow an end-to-end approach, employing different data proportions at various stages. In accordance with Eqs. (10) and (14), the signals received by the array are processed, resulting in data with dimensions of $2M\times L$. These data are then converted to the real number domain: ${X(f)}_{[2M,L]}^{C} \rightarrow Y(f)_{[2M \times L,1]}^{R}$, where *C* represents the complex field and *R* represents the real field. Since the fully connected layer model employs a classification method to output the probability $P_{Y(\hat{w})}$ of best matching the real direction angle, the estimated direction angle is determined by matching the probability-based index positions with the real direction angles. The result is the value corresponding to the highest probability, serving as the estimated direction angle. Here, $\theta \in [{0}^{\circ },{180}^{\circ }]$. The estimation process and the overall design of the model algorithm are depicted in Fig. 3.

From Fig. 3, it can be observed that we initially extract key magnitude and phase features from the time-domain signal and transform them into the frequency domain, followed by normalization. Subsequently, we utilize the Bayesian optimization algorithm to explore the hyperparameter space set $\varrho$. The problem of hyperparameter search closely resembles a Bandit problem. Therefore, for resource allocation and scheduling, based on the available experimental environment (as detailed in Table 1), we employ the Asynchronous Successive Halving Algorithm (ASHA)^43^ to expedite task processing. ASHA sets the task combination limit to $\varepsilon$ (where $\varepsilon = {\prod \varrho }$), the maximum job scheduling limit within a single task to $\tau$, and performs asynchronous Successive Halving (SHA) within each task. In each $\tau$, it evaluates all sub-tree base classifiers of AutoMPDNN to minimize the validation error, thus correcting the model’s convergence. The entire process of automatic hyperparameter selection is presented in Algorithm 2.

**Algorithm 2:**
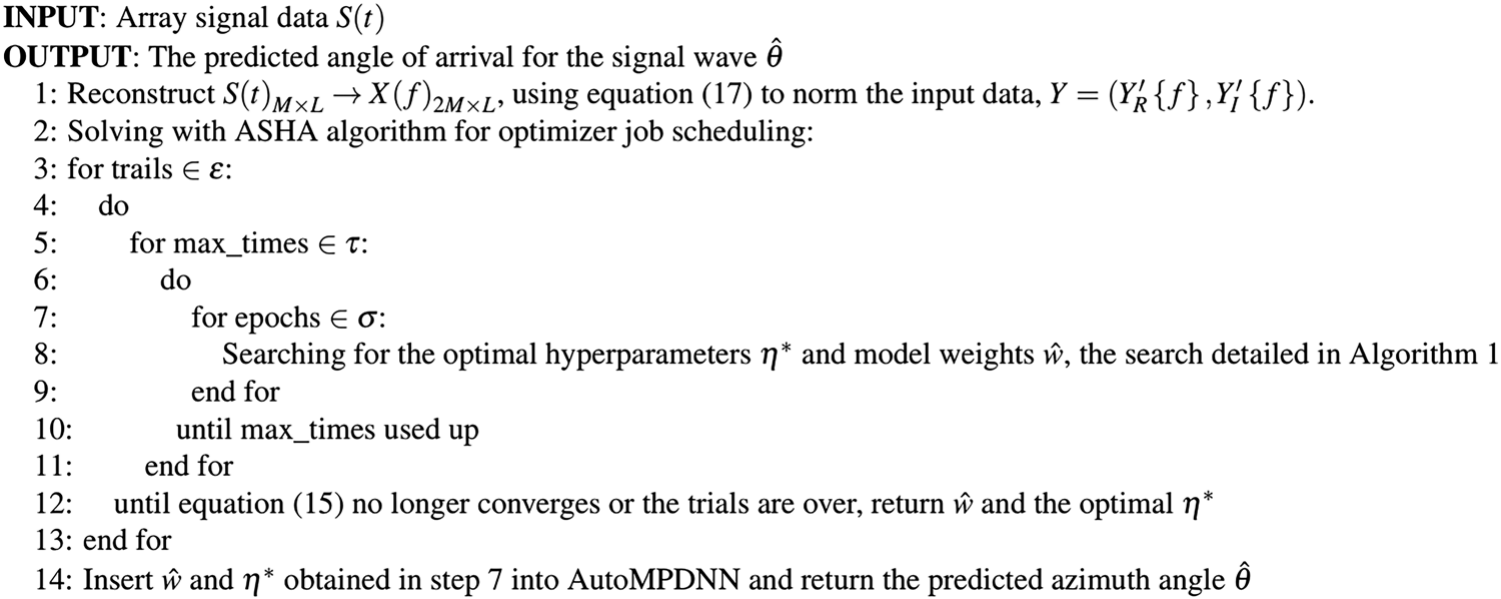
The core process of AutoMPDNN.

## Results

### Experimental environment

The experimental environment settings and coding languages related to this paper are shown in Table 1 (simulated data generation used MATLAB 2019b).

**Table 1:** Experimental environment configuration.

CPU	GPU	System	Code	Memory
13th Gen Intel(R) Core(TM)	NVIDIA GeForce	Windows 11	Python3.8	DDR5
i5-13600K 3.50 GHz	RTX 4070Ti	22621.2283	Torch2.0.0	64GB

In the following simulation experiments, we set the target source frequency to 500 Hz. The wavelength is set to 3 meters, and adjacent sensor elements are spaced at half a wavelength, resulting in an inter-element distance of 1.5 meters. The speed of sound is *c*=1500 m/s. The transmitted source signals are narrowband one-source signals, and the signals received by the array sensors are represented as in Eq. (1). The hardware environment configuration required for the experiments is outlined in the table below. SNR is defined as SNR $= 10\cdot {\lg \left( {Ps/Pn} \right) }$ dB, where *Ps* and *Pn* represent the effective power of the signal and noise, respectively. We divided the dataset into three mutually independent sets: Training set, Validating set, and Testing set. The ratio of Training set to Validating set is set to 4:1. The range of classification angles is $\theta \in [{0}^{\circ },{180}^{\circ }]$, with a ${1}^{\circ }$ interval, resulting in a total of 181 categories. The number of elements *M* is set to 8, 12, 16, 24, and 32 for different groups, each with SNR values of 10 dB (Data1), 20 dB (Data2), and 30 dB (Data3). The details of the dataset partitioning for a single group with a specific number of elements are provided in Table 2.

**Table 2:** Division of simulation experiment datasets.

Data	SNR (dB)	Training set	Validating set	Testing set	Classes
Data1	10	181000	45250	1300	181
Data2	20	181000	45250	1300	181
Data3	30	181000	45250	1300	181

### Search space results

**Figure 4:**
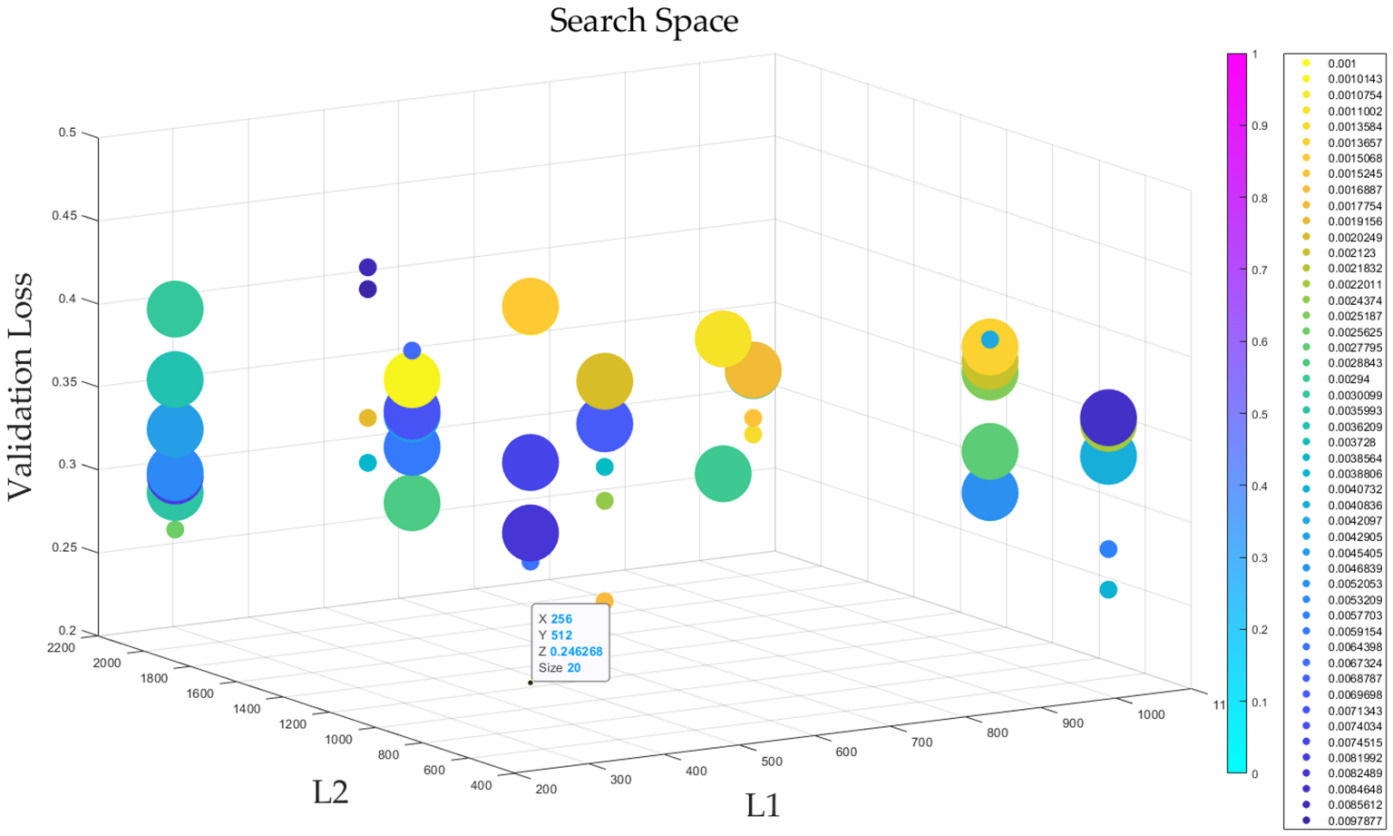
Visualization of hyperparameter space search results of AutoMPDNN. The horizontal axis X represents *l*1, the vertical axis Y represents *l*2, and the Z-axis represents the automated evaluation of validation loss. Each search result is denoted by a circle, with different colors indicating various learning rates, and the circle’s size represents the *batchsize*.

The AutoMPDNN employs the Adam optimizer with 100 epochs. To ensure experimental stability, we conducted 15 runs and averaged the results to obtain the optimal outcome. Employing a supervised learning approach, the Training set data consists of the combined data from Training sets Data1, Data2, Data3. The Validating set is configured in the same manner as the Training set. The hyperparameter search ranges are as follows: *l*1 is chosen from [256, 512, 1024], *l*2 is chosen from [512, 1024, 2048], *lr* ranges from 1e-3 to 1e-2, *p*1 and *p*2 are set as [0.0, 0.1, 0.2] and [0.0, 0.1, 0.2, 0.3] respectively, and *batchsize* is [20, 200, 2000]. In accordance with Eq. (12) and equation (13), we set $\tau$ with max_times=48 and $\sigma$ with trails=12. The reduction factor used to determine the halving rate and amount of SHA is 2. The parameter *ka* for *UCB* is set to 0.25. Validation error serves as the criterion for assessing model convergence. The complete visualization of the hyperparameter space search results on AutoMPDNN is shown in Fig. 4.

Based on the visualization results in Fig. 4 and the output report, it is evident that the optimal results of AutoMPDNN from the hyperparameter space automatic search are *l*1=256, *l*2=512, *lr*=0.001 respectively, both *p*1 and *p*2 are set to 0, and the *batchsize* is 20. The validation error is 0.246268.

### Quantificational analysis results

**Figure 5:**
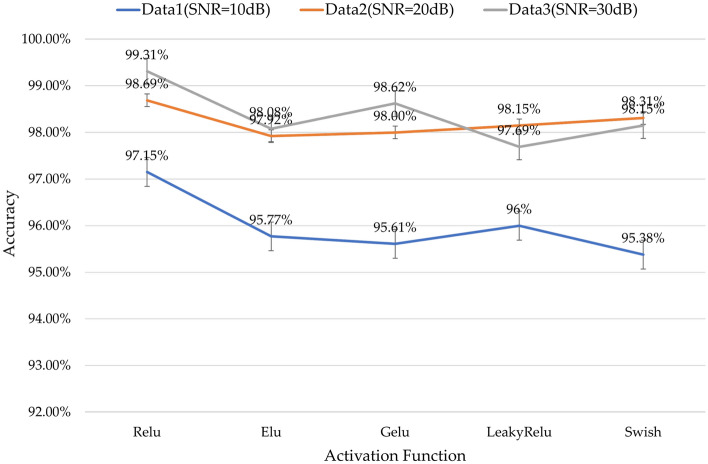
Accuracy comparison chart of different activation functions for num = 16 in AutoMPDNN.

The choice of activation function directly affects the convergence results of AutoMPDNN. Relu suppresses the boundaries with negative values after activation, resulting in sparsity of neuron activation. LeakyRelu^44^ introduces a small positive gradient constant value $$\gamma $$ multiplied by $$O^{p}$$ when the activation output $$O^{p} < 0$$, reducing the sparsity of neurons. Elu^45^ employs a small positive gradient constant value $$\gamma $$ multiplied by $$ ( e ^{O^{p}} - 1 )$$ when the activation output $$O^{p} < 0$$, resulting in a centered approach closer to zero to reduce neuron sparsity. Swish^46^ directly uses $$O^{p}$$ multiplied by the soft gate function $$\frac{1}{1 + e^{- \beta {\cdot O}^{p}}}$$, where $$\beta $$ is a fixed hyperparameter, and the Swish activation function more strictly controls the negative activation states as they approach zero and diverge from zero. Gelu^47^ multiplies $$O^{p}$$ by $$P\left( x \le O^{p} \right) $$, where *P* is the cumulative distribution function of the Gaussian distribution. Gelu offers more flexible precision at the gate boundaries. To select the most suitable activation function for AutoMPDNN, we first fix the number of elements at num = 16. We then compare different activation functions, including LeakyRelu, Elu, Swish, Gelu, and Relu under various SNR Data conditions in our experiments. The results of their prediction accuracy are shown in Fig. 5.

From Fig. 5, it can be observed that Relu exhibits strong suppression of negative values in the simulated data of this experiment, providing AutoMPDNN with better sparse data fitting capabilities. In the test datasets at Data1 (SNR = 10 dB), Data2 (SNR = 20 dB), and Data3 (SNR = 30 dB), the test accuracy can reach up to 99.31%, 98.69%, and 97.15%, making it more suitable for AutoMPDNN compared to other activation functions.

**Figure 6:**
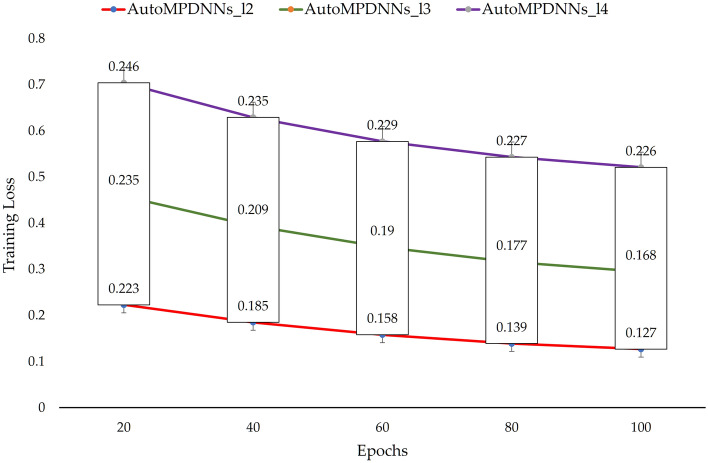
Comparison of different numbers of layers for num = 16 and convergence of training loss with different epochs.

**Figure 7:**
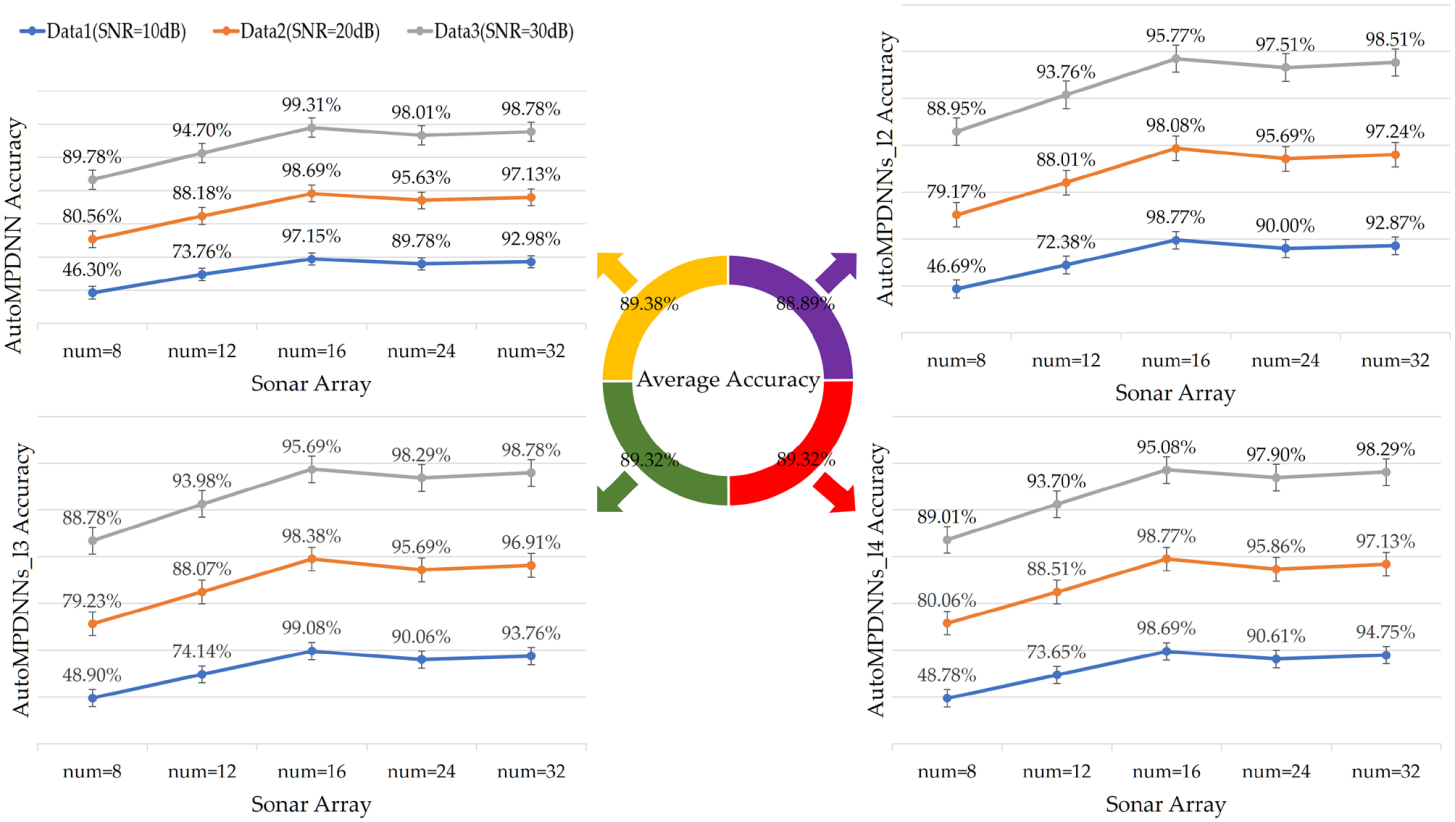
Comparison of different number of array elements (*M* = 8, 12, 16, 24, 32) at various Datas Groups (X-axis: the number of elements).

In deep learning, the depth and width of a network are often critical factors^48^ limiting the performance of deep learning models. To assess the impact of network depth and neuron width on model prediction accuracy, we increased the number of hidden layers in AutoMPDNN to *l*=3 and *l*=4. For *l*=3, the intermediate layer contained 512 neuron nodes, while for *l*=4, it consisted of 512*3 and 1024 neuron nodes, with 512 neuron nodes in all layers except the last one. The dropout rate was set to 0.1 for all layers except the first, where it was set to 0. The models are denoted as AutoMPDNNs_l3 and AutoMPDNNs_l4, respectively. For comparison, we expanded the number of neuron nodes in two layers to 512, and the dropout settings remained the same, referred to as AutoMPDNNs_l2. We train, validate, and test AutoMPDNNs_ln (n $=$ 2,3,4) using the same strategy as AutoMPDNN, but with some differences in other aspects. Through controlled variable analysis, the training errors and experimental prediction results using the hyperparameter search algorithm are shown as Figs. 6 and 7 (the column of num = 16).

From Figs. 6 and 7 (the column of num = 16), it is evident that AutoMPDNNs_l2 achieves higher accuracy at SNRs of Data1 (SNR = 10 dB), Data2 (SNR = 20 dB), and Data3 (SNR = 30 dB), with accuracies of 98.77%, 98.08%, and 95.77%, respectively. This performance is superior to that of AutoMPDNNs_l3 and AutoMPDNNs_l4, with AutoMPDNNs_l2 also exhibiting the best training convergence. However, it should be noted that the overall accuracy is lower for AutoMPDNN compared to AutoMPDNNs_ln (n $=$ 2,3,4). In summary, AutoMPDNN demonstrates better overall performance with *M*=16 array elements.

Under the optimal parameter conditions established for different layer numbers and neuron node counts in the search space, we conducted experiments to analyze the accuracy in various environmental conditions at different SNRs. The results are presented in Fig. 7.

From the vertical comparison in Fig. 7, it is evident that AutoMPDNN achieved accuracy rates of 88.17% (SNR = 10 dB), 94.66% (SNR = 20 dB), and 97.45% (SNR = 30 dB) when the array size was equal to or greater than 12, with an average accuracy of 93.43%. AutoMPDNNs_l2 achieved accuracy rates of 80.14% (SNR = 10 dB), 91.64% (SNR = 20 dB), and 94.90% (SNR = 30 dB) when the array size was equal to or greater than 12, with an average accuracy of 88.89%. AutoMPDNNs_l3 achieved accuracy rates of 81.1% (SNR = 10 dB), 91.66% (SNR = 20 dB), and 95.10% (SNR = 30 dB) when the array size was equal to or greater than 12, with an average accuracy of 89.32%. AutoMPDNNs_l4 achieved accuracy rates of 81.30% (SNR = 10 dB), 92.07% (SNR = 20 dB), and 94.80% (SNR = 30 dB) when the array size was equal to or greater than 12, with an average accuracy of 89.39%. AutoMPDNN demonstrated the best generalization performance.

### Qualitative analysis results

Additionally, the cost and test inference speed for AutoMPDNN and AutoMPDNNs_ln (n $=$ 2,3,4) are detailed in Table 3. We compared evaluation metric, mean accuracy, target number, degree resolution and measurement strategy for single snapshots, as shown in Table 4, with a comparison to current mainstream deep learning models. AutoMPDNN achieved a mean accuracy within a ${1}^{\circ }$ estimation error range, reaching 98.38%.

**Table 3:** Inference speed and cost of the proposed network model.

	GPU Usage (GB)	Training time (h)	Model parameter size (MB)	Total model parameters	Model size (MB)	Inference speed (ms)
AutoMPDNN	1.103	8	0.89	232885	1.77	0.3131
AutoMPDNNs_l2	1.122	8.5	1.42	372405	2.84	0.3552
AutoMPDNNs_l3	1.140	9	2.42	635061	4.84	0.3682
AutoMPDNNs_l4	1.155	10	4.87	1253045	9.56	0.3895

**Table 4:** Single snapshot comparison with other neural networks.

	Evaluation metric	Mean accuracy	Target number	Degree resolution	Measurement strategy
FNN^24^	Cross-Entropy Loss Function	95.9%	2	${1}^{\circ }$	Classification
CNN^24^	Cross-Entropy Loss Function	74.1%	2	${1}^{\circ }$	Classification
NN^23^	Mean Squared Error	Spatial beam (SNR = 20–40 dB)	1	${0.001}^{\circ }$	Regression
Resnet^25^	Root Mean Squared Error	90.1%(SNR = 30 dB)	2	${1}^{\circ }$	Classification
DeepMLE^26^	Root Mean Squared Error	90.1%(SNR = 30 dB)	2	${1}^{\circ }$	Classification
DeepDOA^27^	Root Mean Squared Error	spatial beam	1–3	${0.001}^{\circ }$	Regression
DeepMPDR^28^	Mean Squared Error	100%	1	$\ge {4}^{\circ }$	Classification
Random forests_10^40^	Gini Coefficient	87.78%	1	${1}^{\circ }$	Classification
Random forests_50^40^	Gini Coefficient	89.43%	1	${1}^{\circ }$	Classification
Random forests_100^40^	Gini Coefficient	89.79%	1	${1}^{\circ }$	Classification
AutoMPDNN	Cross-Entropy Loss Function	98.38%	1	${1}^{\circ }$	Classification
AutoMPDNNs_l2	Cross-Entropy Loss Function	97.54%	1	${1}^{\circ }$	Classification
AutoMPDNNs_l3	Cross-Entropy Loss Function	97.71%	1	${1}^{\circ }$	Classification
AutoMPDNNs_l4	Cross-Entropy Loss Function	95.51%	1	${1}^{\circ }$	Classification

**Figure 8:**
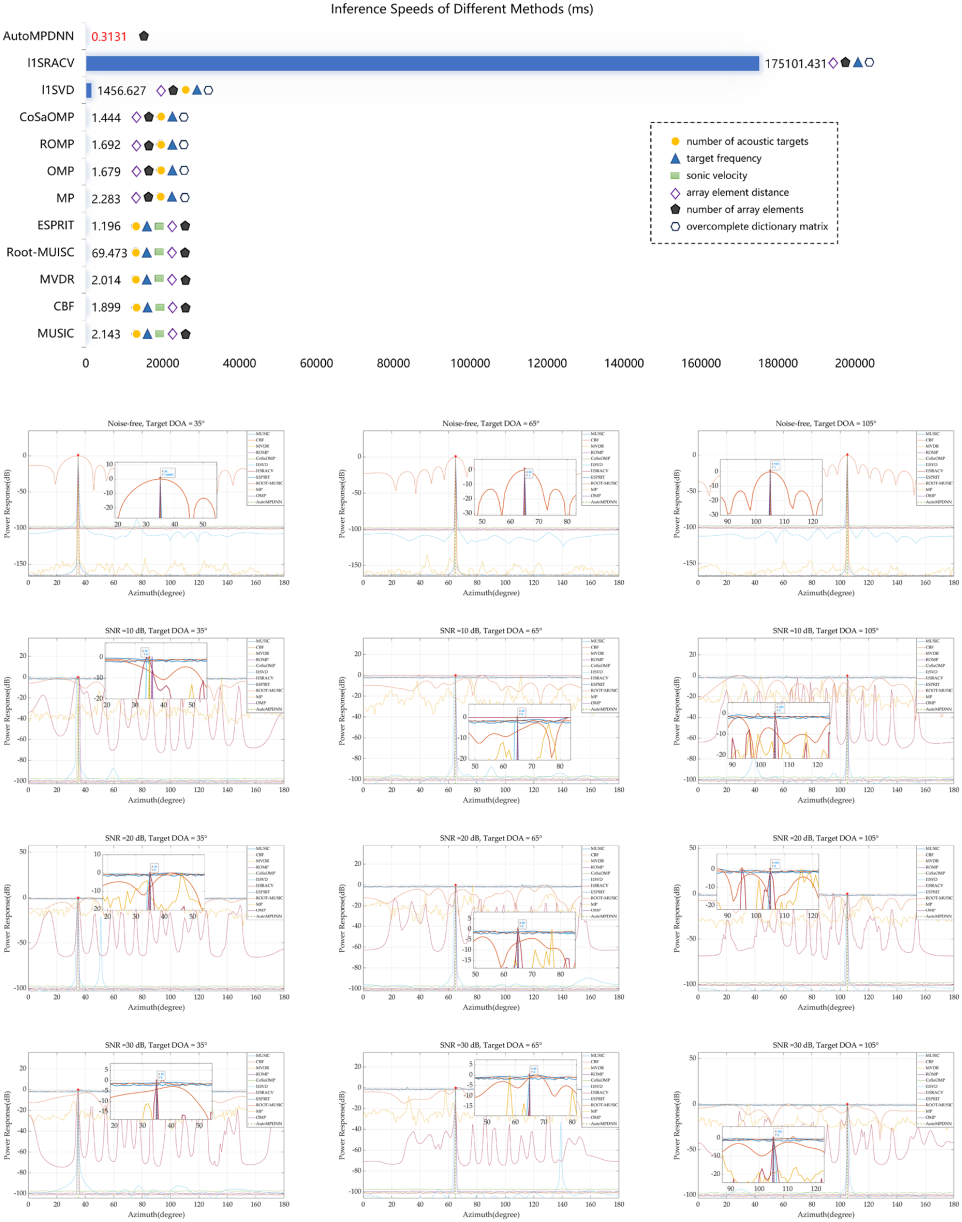
Comparing AutoMPDNN with other classical algorithms and compressive sensing algorithms, the first row shows the inference speed of the algorithm and its required prior conditions. The last three rows show the simulation DOA results for a single snapshot and one array element at ${35}^{\circ }$, ${65}^{\circ }$ and ${105}^{\circ }$, respectively, under SNR levels of 10 dB, 20 dB, 30 dB.

We further compared the resolution accuracy and inference speed of AutoMPDNN with traditional DOA estimation methods in Fig. 8. We selected real azimuth angles of ${35}^{\circ }$, ${65}^{\circ }$, and ${105}^{\circ }$ for comparison, evaluating the performance of AutoMPDNN against classical and compressed aware sparse DOA algorithms at array element num = 16 of Data1 (SNR = 10 dB), Data2 (SNR = 20 dB), Data3 (SNR = 30 dB) in Fig. 8.

The results in Fig. 8 indicate that as SNR gradually decreases, the MUSIC, ROOT-MUISC and ESPRIT algorithm exhibit severe distortions in predicting azimuth angles due to data sparsity, leading to the appearance of multiple false peaks. The CBF method, because of sparse data, exhibits significant phase compensation errors, while MVDR tends to experience distortions when inverting the covariance matrix. Although sparse reconstruction algorithms can achieve high accuracy, they require a large number of prior conditions. Among them, l1SVD and l1SRACV require much more time than other algorithms due to the complexity of computation. AutoMPDNN not only maintains high super-resolution (The predictive results of AutoMPDNN are represented by the dashed green lines with red starer) but also has the fastest real-time inference time. Under the conditions of sparse single-snapshot samples, both of these algorithms demonstrate blurry and inaccurate resolution. However, AutoMPDNN continues to provide accurate azimuth angle estimation even in scenarios with single snapshot.

## Conclusion

In this paper, we propose an AutoMPDNN and its variants AutoMPDNNs_ln for one-source localization under single-snapshot conditions. This model estimates the DOA of underwater acoustic signals end-to-end by learning amplitude and phase difference information. To ensure the network model’s generalization and global optimality, we introduce a dropout layer and an automated parameter learning algorithm based on Bayesian optimization to constrain the network model’s parameter space. We automatically search for algorithm hyperparameters within the parameter space to achieve the best convergence performance under different SNR conditions. AutoMPDNN overcomes the strong data dependency of traditional methods in single snapshot-sampling. We conduct a comprehensive comparison of AutoMPDNN with other deep learning models from both horizontal and vertical perspectives. The analysis and comparison results on different simulated experimental data demonstrate the efficiency and effectiveness of AutoMPDNN.

## Data Availability

Data will be made available on request. If you need the data of this paper, please contact the author Bin Zhang (email: zhangbin9145@stu.ouc.edu.cn).

## References

[1] Karim, B. A. & Ali, H. K. Computationally efficient MUSIC based DOA estimation algorithm for FMCW radar. *J. Electron. Sci. Technol.* **21**(1), 46–64 (2023).

[2] Zhou, L., Ye, K., Qi, J., Hong, S. & Sun, H. Underwater DOA estimation based on cross-correlation domain for relocating improved nested array. *Digit. Signal Process.* **128**, 103606 (2022).

[3] Bhogavalli, S., Hari, K., Grivel, E. & Corretja, V. Estimating the target DOA, range and velocity using subspace methods in a MIMO OFDM DFRC system. *Signal Process.* **209**, 109007 (2023).

[4] Van Veen, B. D. & Buckley, K. M. Beamforming: A versatile approach to spatial filtering. *IEEE ASSP Mag.* **5**(2), 4–24 (1988).

[5] Capon, J. High-resolution frequency-wavenumber spectrum analysis. *Proc. IEEE* **57**(8), 1408–1418 (1969).

[6] Knapp, C. & Carter, G. The generalized correlation method for estimation of time delay. *IEEE Trans. Acoust. Speech Signal Process.* **24**(4), 320–327 (1976).

[7] Schmidt, R. Multiple emitter location and signal parameter estimation. *IEEE Trans. Antennas Propag.* **34**(3), 276–280 (1986).

[8] Roy, R. & Kailath, T. ESPRIT-estimation of signal parameters via rotational invariance techniques. *IEEE Trans. Acoust. Speech Signal Process.* **37**(7), 984–995 (1989).

[9] Rao, B. D. & Hari, K. V. S. Performance analysis of Root-Music. *IEEE Trans. Acoust. Speech Signal Process.* **37**(12), 1939–1949 (1989).

[10] Liu, B., Gui, G., Matsushita, S.-Y. & Xu, L. Adaptive filtering algorithm for direction-of-arrival (DOA) estimation with small snapshots. *Digit. Signal Process.* **94**, 84–95 (2019).

[11] Fortunati, S., Grasso, R., Gini, F., Greco, M. S. & LePage, K. Single-snapshot DOA estimation by using compressed sensing. *EURASIP J. Adv. Signal Process.* **2014**, 1–17 (2014).

[12] Ma, Y., Cao, X., Wang, X., Greco, M. S. & Gini, F. Multi-source off-grid DOA estimation with single snapshot using non-uniform linear arrays. *Signal Process.* **189**, 108238 (2021).

[13] Fang, Y., Zhu, S., Zeng, C., Gao, Y. & Li, S. DOA estimations with limited snapshots based on improved rank-one correlation model in unknown nonuniform noise. *IEEE Trans. Veh. Technol.* **70**(10), 10308–10319 (2021).

[14] Zeng, C., Zhu, S., Li, S., Liao, Q. & Wang, L. Sparse frame DOA estimations via a rank-one correlation model for low SNR and limited snapshots. *Appl. Comput. Harmon. Anal.* **41**(2), 362–383 (2016).

[15] Candes, E. J., Romberg, J. & Tao, T. Robust uncertainty principles: exact signal reconstruction from highly incomplete frequency information. *IEEE Trans. Inf. Theory* **52**(2), 489–509 (2006).

[16] Marques, E. C., Maciel, N., Naviner, L., Cai, H. & Yang, J. A review of sparse recovery algorithms. *IEEE Access.* **7**, 1300–1322 (2019).

[17] Wang, J., Chen, L. & Yin, Z. Array signal MP decomposition and its preliminary applications to DOA estimation. In *Intelligent Control and Automation: International Conference on Intelligent Computing* (*ICIC*), 54–59 (Springer, 2006).

[18] Emadi, M., Miandji, E. & Unger, J. OMP-based DOA estimation performance analysis. *Digital Signal Process.* **79**, 57–65 (2018).

[19] Needell, D. & Vershynin, R. Signal recovery from incomplete and inaccurate measurements via regularized orthogonal matching pursuit. *IEEE J. Sel. Top. Signal Process.* **4**(2), 310–360 (2010).

[20] Needell, D. & Tropp, J. A. CoSaMP: Iterative signal recovery from incomplete and inaccurate samples. *Appl. Comput. Harmon. Anal.* **26**(3), 301–321 (2009).

[21] Malioutov, D., Cetin, M. & Willsky, A. S. A sparse signal reconstruction perspective for source localization with sensor arrays. *IEEE Trans. Signal Process.* **53**(8), 3010–3022 (2005).

[22] Yin, J. & Chen, T. Direction-of-Arrival estimation using a sparse representation of array covariance vectors. *IEEE Trans. Signal Process.* **59**(9), 4489–4493 (2011).

[23] Weiß, M., Kohler, M., Saam, A. & Worms, J. Single snapshot DoA estimation from a Rotman lens using machine learning techniques. In *2020 21st International Radar Symposium* (*IRS*), 35–39 (IEEE, 2020).

[24] Ozanich, E., Gerstoft, P. & Niu, H. A feedforward neural network for direction-of-arrival estimation. *J. Acoust. Soc. Am.* **147**(3), 2035–2048 (2020). 32237833 10.1121/10.0000944

[25] Lima de Oliveira, M. L. & Bekooij, M. J. G. ResNet applied for a single-snapshot DOA estimation. In *2022 IEEE Radar Conference* (*RadarConf22*), 1–6 (IEEE, 2022).

[26] de Oliveira, M. L. L. & Bekooij, M. J. G. Deep-MLE: Fusion between a neural network and MLE for a single snapshot DOA estimation. In *2022 IEEE International Conference on Acoustics, Speech and Signal Processing* (*ICASSP2022*), 3673–3677 (IEEE, 2022).

[27] Yang, Z., Chen, P., Geng, R. & Jia, Y. DeepDOA: A novel deep learning-based method for DOA superresolution in a single snapshot. In *2022 IEEE 5th International Conference on Electronics Technology* (*ICET*), 703–706 (IEEE, 2022).

[28] Zheng, R., Sun, S., Liu, H., Chen, H. & Li, J. Interpretable and efficient beamforming-based deep learning for single snapshot DOA estimation. *IEEE Sens. J.* (2023).

[29] Arık, S. O. *et al*. Deep voice: Real-time neural text-to-speech. In *International Conference on Machine Learning*, 195–204 (PMLR, 2017).

[30] Szegedy, C. *et al*. Going deeper with convolutions. In *Proceedings of the IEEE Conference on Computer Vision and Pattern Recognition*, 1–9 (IEEE, 2015).

[31] Zhang, H. *et al*. Deep adaptive AEC: Hybrid of deep learning and adaptive acoustic echo cancellation. In *ICASSP 2022-2022 IEEE International Conference on Acoustics, Speech and Signal Processing* (*ICASSP*), 756–760 (IEEE, 2022). 10.1109/icassp43922.2022.9747445 PMC12045126 40313329

[32] Devlin, J., Chang, M.-W., Lee, K. & Toutanova, K. Bert: Pretraining of deep bidirectional transformers for languageunderstanding. arXiv preprint arXiv:1810.04805 (2018).

[33] Griffin, D. & Lim, J. Signal estimation from modified short-time Fourier transform. *IEEE Trans. Acoust. Speech Signal Process.* **32**(2), 236–243 (1984).

[34] Bahdanau, D., Cho, K. & Bengio, Y. Neural machine translation by jointly learning to align and translate. arXiv preprint arXiv:1409.0473 (2014).

[35] Vaswani, A. *et al*. Attention is all you need. arXiv preprint arXiv:1706.03762 (2017).

[36] Rumelhart, D. E., Hinton, G. E. & Williams, R. J. Learning representations by back-propagating errors. *Nature* **323**(6088), 533–536 (1986).

[37] Guo, M.-H., Liu, Z.-N., Mu, T.-J. & Hu, S.-M. Beyond self-attention: External attention using two linear layers for visual tasks. *IEEE Trans. Pattern Anal. Mach. Intell.* **45**(5), 5436–5447 (2022). 10.1109/TPAMI.2022.3211006 36197869

[38] Hahnloser, R. H., Sarpeshkar, R., Mahowald, M. A., Douglas, R. J. & Seung, H. S. Digital selection and analogue amplification coexist in a cortex-inspired silicon circuit. *Nature* **405**(6789), 947–951 (2000). 10879535 10.1038/35016072

[39] Hinton, G. E., Srivastava, N., Krizhevsky, A., Sutskever, I. & Salakhutdinov, R. R. Improving neural networks by preventing co-adaptation of feature detectors. arXiv preprint arXiv:1207.0580 (2012).

[40] Breiman, L. Random forests. *Mach. Learn.* **45**, 5–32 (2001).

[41] Garner, S. R. *et al*. Weka: The waikato environment for knowledge analysis. In *Proceedings of the New Zealand Computer Science Research Students Conference*, Vol. 1995, 57–64 (Citeseer, 1995).

[42] Snoek, J., Larochelle, H. & Adams, R. P. Practical Bayesian optimization of machine learning algorithms. In *Advance Neural Information Processing Systems*, **25** (2012).

[43] Li, L. *et al*. A system for massively parallel hyperparameter tuning. arXiv preprint arXiv:1810.05934 (2018).

[44] Maas, A. L., Hannun, A. Y., Ng, A. Y. & others. Rectifier nonlinearities improve neural network acoustic models. In *Proceedings of ICML* Atlanta, GA, Vol. 30, 3 (2013).

[45] Clevert, D.-A., Unterthiner, T. & Hochreiter, S. Fast and accurate deep network learning by exponential linear units (elus). arXiv preprint arXiv:1511.07289 (2015).

[46] Ramachandran, P., Zoph, B. & Le, Q. V. Searching for activation functions. arXiv preprint arXiv:1710.05941 (2017).

[47] Hendrycks, D. & Gimpel, K. Gaussian error linear units (gelus). arXiv preprint arXiv:1606.08415 (2016).

[48] Delalleau, O. & Bengio, Y. Shallow vs. deep sum-product networks. In *Advance Neural Information Processing Systems*, **24** (2011).

