# Author Correction: An automated multi-layer perceptron discriminative neural network based on Bayesian optimization achieves high-precision one-source single-snapshot direction-of-arrival estimation

**DOI:** 10.1038/s41598-024-65500-8

**Published:** 2024-06-27

**Authors:** Bin Zhang, Jiawen He, Peishun Liu, Liang Wang, Ruichun Tang

**Affiliations:** 1 Department of Computer Science and Technology, Ocean University of China, Qingdao, 266100 China; 2 Department of Marine Technology, Ocean University of China, Qingdao, 266100 China

Correction to: *Scientific Reports* 10.1038/s41598-024-60798-w, published online 05 May 2024

The original version of this Article contained an error in Algorithm 2, where the pseudocode displayed an incorrect reference to Table 1.

The incorrect Algorithm 2 and the accompanying legend appear below.

**Algorithm 2:**
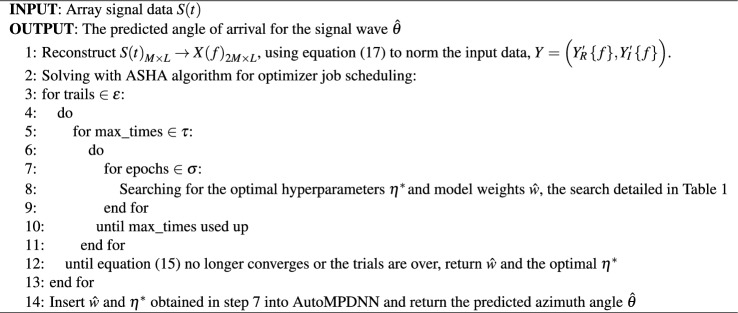
The core process of AutoMPDNN.

The original Article has been corrected.

